# Invalids and Exertive Exercise

**Published:** 1854-02

**Authors:** 


// HALL'S JOURNAL OF HEALTH.

VOL. I.] FEBRUARY, 1854. [NO. II.

### INVALIDS AND EXERTIVE EXERCISE.

CONTINUED FROM LAST NUMBER.

With all this, and looking like the ruddiest specimen of health in the country round about, I am still (you will be comforted to hear) troubled occasionally with my sleep-robber of a cough; and, in Boston, the other day, on breathing that essence of pepper and icicles which they call their "East Wind," I was seized with the old hemorrhage of the lungs, and bled myself weak again. But I rallied immediately on returning to this Highland air, and am well once more—as well, that is to say, as is consistent with desirable nervous susceptibility. The kiss of the delicious South Wind of to-day, (November 30,) would be half lost upon the cheek of perfect health.

I fear I cannot sufficiently convey to you my sense of the importance of *a horse* to an invalid. In my well-weighed opinion, ten miles a day in the saddle would cure more desperate cases, (particularly of consumption,) than all the changes of climate and all the medicines in the world. It is vigorous exercise without fatigue. The peculiar motion effectually prevents all irritation of cold air to the lungs, on the wintriest day. The torpid liver and other internal organs are more shaken up and vivified by the trot of a mile than by a week of feeble walking. The horse (and you should own and love him) is company enough, and not too much. Your spirits are irresistibly enlivened by the change of movement and the control of the animal. Your sense of strength and activity, (in which lies half the self-confidence as to getting well, which the doctors think so important,) is plus one horse. With the difference from walking, as to pulling upon the forces of the spine and consequently upon the brain, it is recommended by the best English physicians as much the preferable exercise for men of intellectual pursuits. And, last, (I think not least,) the lungs of both body and soul are expanded by the daily consciousness of inhabiting a larger space —by having an eagle's range rather than a snail's—by living a life which occupies ten miles square of the earth's surface, rather than that "half mile" which you speak of as the extent of your daily walk. The cost is trifling. At this particular season, when horses are beginning, as they say at the livery stables, to "eat their heads off," you may buy the best you can want for fifty dollars, and his feed costs thirty cents a day. As the horse and the doctor are seldom necessities of one and the same man, you may rather find it an economy—apothecary and all.

In that "majority" I have spoken of above, there are, (as in all majorities,) some voters of not much consequence individually, but still worth keeping an eye upon. Briefly to name one or two:—There are so few invalids who are invariably and conscientiously *untemptable* by those deadly domestic enemies, *sweetmeats, pastry* and *gravies,* that the usual civilities at a meal are very like being politely assisted to the grave. The care and nurture of the *skin* is a matter worth some study; for it is capable not only of being negatively healthy, but positively luxurious in its action and sensations—as every well-groomed horse knows better than most men. The American *liver* has a hard struggle against the greasy cookery of our happy country. The impoverished *blood* of the invalid sometimes requires that "glass of wine for the stomach's sake" recommended by the Apostle. Just *sleep* enough and just *clothing* enough are important adjustments, requiring more thought and care than are usually given to them. For a little philosophy in your habitual *posture* as you sit in your chair, your *lungs* would be very much obliged to you. An analysis of the *air* we live and sleep in, would be well worth looking into occasionally. And there are two things that turn sour in a man, without constant and sufficient occupation upon something beside the domestic circle—the *temper* and the *ambition.* * *

Thus much, of my reply to our clerical fellow-sufferer, may interest you, dear invalid reader. Of the medicine of "Out-doors at Idlewild"—the mingled salubrity of the climate of mountain and river around us—I should have said more to one unanchored in a home and a parish. From one who writes so frankly and sensibly as he, we must hope to hear again, however, and, with another opportunity, I may again ask for invalid indulgence, and return to the theme.

In a future number I purpose giving an illustration of the truthfulness, in the main, of the statements made above, showing in a more remarkable manner what may be accomplished by medical advice, without medicine, when the patient is willing to get well without medicine.

I wish here to take exception to the clergyman's language—"By the advice of physicians here and in New York, I spent two winters at the South * * * * but got no essential benefit

* * * neglecting further medical advice, I bought, two years since, a pleasant site for a country residence * * * * * but I don't get well." There is a want of courtesy in the manner in which his medical advisers are spoken of, which is unbecoming a clergyman, and especially so, if that advice was gratuitous, as is most probably the case. After discarding physicians and prescribing for himself for two years, it seems, from his own admission, that he is not well. Nor would any man ever get well by pursuing a course so irrational and absurd as that which this clergyman adopted. It is, however, an illustration of the futility of simply going to the sunny South to get rid of a cough, and how much may be done by remaining in the North and pursuing a proper course, under medical advice, as in the case of the gentleman to whom the clergyman wrote.

